# Structural Balance of Opinions

**DOI:** 10.3390/e23111418

**Published:** 2021-10-28

**Authors:** Malgorzata J. Krawczyk, Krzysztof Kułakowski

**Affiliations:** Faculty of Physics and Applied Computer Science, AGH University of Science and Technology, Mickiewicza 30, PL-30059 Kraków, Poland; malgorzata.krawczyk@agh.edu.pl

**Keywords:** Heider balance, nonlinear dynamics, American states, comfort hypothesis

## Abstract

The concept of Heider balance, usually applied to interpersonal relations, is generalized here to opinions gathered in surveys. At first, we compare four algorithms, which drive a matrix dataset to a balanced state. The criterion is that the final state obtained with an algorithm should be as close as possible to the initial state. The result is that deterministic differential equations work better than their Monte Carlo counterparts. Next, we apply the winning algorithms to the matrix of correlations between opinions gathered in American states between 1974 and 1998. The results are interpreted in terms of the classic comfort hypothesis (E. Babbie, 2007).

## 1. Introduction

The modeling of dynamical processes is at the forefront of research in numerous branches of science, including political science [1], sociology [2] and social psychology [3]. The aim of modeling a social system is not to reproduce it with its whole complexity, but rather to postulate causal relations which could be next confronted with statistical observations on selected aspects of the system [4]. Here, we are interested in consequences of the process of removal of cognitive dissonance for the characteristics of opinions, collected in social surveys. Our aim is to present and discuss a computational scheme, which seems appropriate here.

According to Andrew Heywood ([5], p. 217), there are four rival models of voting: the party identification model, the sociological model, the rational choice model and the dominant ideology model. In a nutshell, the first three models explore the attachments of voters to political parties, social classes or their personal self-interests, respectively. The fourth model admits that voter behavior is shaped by ideological manipulation through education and mass media. This perspective has been well established in political sciences since the famous essay by Philip E. Converse in 1964 [6,7]. As it is argued therein, the majority of voters are unable to analyze political facts abstractly. Instead, they make decisions basing upon rough associations related to ideology. The process of reaching decisions was investigated also in more general frames by Cristina Bicchieri ([8], p. 4): apart from the rational path, individuals can associate a given situation with their past experience and react almost subconsciously, basing upon such a categorization. Our view here is that these ideologies and categorizations, even if irrational, should be internally consistent.

A specific kind of consistency—with respect to friendly and hostile interpersonal relations—is known in social psychology under the name of Heider (or structural) balance (HB) [9,10,11,12]. In a fully connected social network, states which are balanced in this sense consist of two subsets of nodes: internally friendly but mutually hostile. In such a state, every triad (three nodes and three relations between them) contains either two or zero hostile relations. Balanced states are commonly interpreted as being devoid of cognitive dissonance [13], which is perceived by an actor at a node if any of the following rules [14] are broken:

- A friend of my friend is my friend;
- An enemy of my friend is my enemy;
- A friend of my enemy is my enemy;
- An enemy of my enemy is my friend.

In its earliest formulation [9], the concept of HB was applied to relations of actors to actors and objects. Soon, this application was expanded to relations that are exclusively interpersonal [11,12,15,16,17], and to relations between political entities, such as governments of different states [18,19,20,21,22,23]. Our goal here is to apply HB to statements, which are answered in interviews about political issues. We postulate two statements to be ’friendly’ if they are positively correlated in the interviews. In this way, we intend to gain knowledge about the consistency of given sets of statements as perceived by interviewees.

We should not expect that the answers will be logically consistent in our perspective, as the perspective of our interviewees is different from ours. Neither should we expect that the collected material will be already equivalent to a balanced state. Then, our first task will be to find an algorithm which is optimal, in the sense that it drives the system to a balanced state that is as close as possible to the initial state. A final state obtained with this algorithm should be simultaneously (i) balanced, then consistent; and (ii) only minimally distorted with respect to the initial state. Having the algorithm, we intend to apply it to data from interviews, available in the literature. The goal will be to identify statements that co-occur. We expect to gain insight to portraits of voters: sets of statements which seem to them to be consistent.

We underline that this program of research is most coherent with the dominant ideology model, the last out of the four models specified by Heywood [5]. In the other three, one needs information about the structure of parties, classes or interests of voters. Consequently, any algorithm should be able to make use of these data, including realistic values of numerous parameters. Such an approach is out of the scope of this paper. On the other hand, the binomial character of relations in any balanced state is a natural consequence of a white–black view of the world. Therefore we consider the Heider balance to be properly framed within the model of dominant ideology. Finally, to identify a group of enemies is known to be the best method to consolidate one’s own group. As was famously stated by Alexis de Tocqueville, *In politics, shared hatreds are almost always the basis of friendships* ([24], p. 73). It is here where the fourth rule of an enemy of one’s own enemy applies.

We note that the most prominent application of the Monte Carlo method to locate the minima of a work function is the algorithm of simulated annealing [25]. The target there was to locate a global minimum, the deepest of all of them. Here, our aim is different: we search for a local minimum that is most close to the initial state in the space of states. The issue that the minima are of the same or different depth is of secondary importance here.

In the next section, we report four algorithms known in the literature, which lead a fully connected network to a balanced state. Section III is meant to describe the set of data, taken from literature [26]. In Section IV, we compare the performance of the algorithms, and we apply the selected one to this dataset. The last section is devoted to discussion.

## 2. Algorithms

There are four algorithms which lead a complete graph to a balanced state. Two of them are discrete, i.e. they operate on signed links: a link $s_{ij}$ between nodes *i* and *j* can be positive or negative, $s_{ij}=\pm 1$ [18]. Both of these algorithms are based on the Monte Carlo method. The next two algorithms operate on real numbers ($s_{ij}\in <-1;+1>$), and they are deterministic [27]. The algorithms can be briefly described as follows.

In the first algorithm, termed local triad dynamics (LTD) [18,28], a triad of nodes is selected randomly. If the triad is balanced, it remains unchanged. If it contains three negative links, the sign of one of them is switched to be positive. If one link is negative, it is switched to be positive with probability *p*, or one of two positive links is switched to be negative with probability 1−p. Next, another triad is updated, and so on. Once all triads are balanced, the algorithm is stopped. It is clear that each balanced state is absorbing. Yet, under LTD, the total number *F* of imbalanced triads can temporarily increase.

In the second algorithm, termed constrained triad dynamics (CTD) [18,28], a link is selected randomly, and an attempt is made to change its sign. If this leads to a decrease in *F*, the change remains; if *F* increases, the change is withdrawn; if *F* is not changed, the change of the link remains with probability 1/2. It is clear that under CTD, *F* cannot increase. Yet the system can be trapped in an unbalanced state, commonly termed “jammed”.

The third algorithm (let us call it DE—differential equations) relies on a set of L=N(N−1)/2 equations, with one per each link:

(1) $$\frac{ds_{ij}}{dt}=\left(1-s_{ij}^{2}\right)\sum_{k=1}^{N-2}s_{ik}s_{kj}$$

where *N* is the total number of nodes. Here, jammed states are possible, too [29]. As each configuration of links $s_{ij}=\pm 1$ is a fixed point, we use $s_{ij}=\pm \left(1-\varepsilon_{ij}\right)$ as the initial states, where $\varepsilon_{ij}$ is a random number. This setting ensures that the values of the variables $s_{ij}$ remain in the range <−1,1>; once the limit is reached, the time derivative is zero. Numerical experiments show that the results do not change visibly as long as $0.1<\varepsilon_{ij}<0.3$.

The fourth algorithm (let us call it DE’) is a modified version of DE, without the prefactor $\left(1-s_{ij}^{2}\right)$. The set of equations is solved analytically [30]. Its another advantage is that the obtained equations are equivalent to an overdamped motion in a potential:

(2) $$U=-\sum_{ijk}s_{ij}s_{jk}s_{ki}$$

which is minimal in balanced states. However, these equations lead the links $s_{ij}$ to infinity in finite time. Therefore, we apply it in combination with the additional condition that $|s_{ij}|\leq 1$ [31]. This condition is executed numerically: once some variable $s_{ij}$ reaches the value +1 (−1), it is prevented from further increasing (decreasing). The correction $\varepsilon_{ij}$ is applied also here to evade an artificial symmetry, where the same value is assigned to all links.

**Figure 1:**
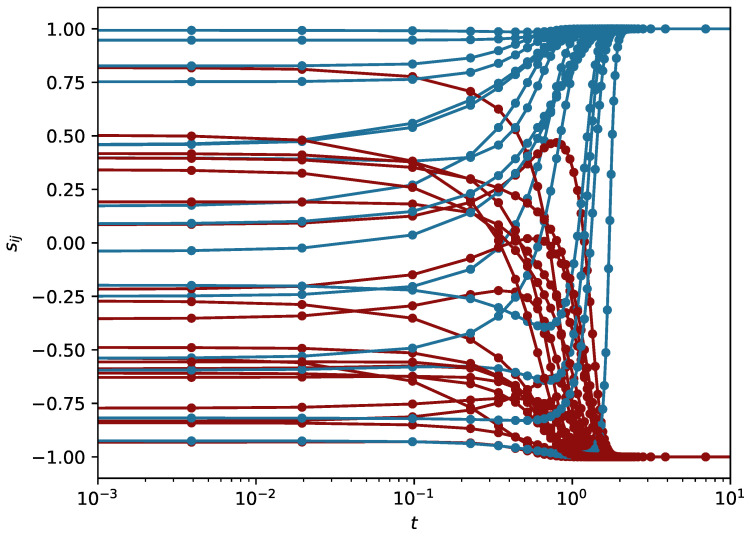
Trajectories $s_{ij}$ dependent on numerical time *t* for N=9, obtained with DE. Colors (online) are added to better distinguish the curves which end at −1 or +1. For the sake of better visibility, the time axis is shown in the logarithmic scale.

For all four algorithms, each balanced state is absorbing; once the system falls there, it remains there. As each triad is balanced, $s_{ij}s_{jk}s_{ki}=+1$, and the energy *U* is of minimal value. Both LTD and CTD remain inactive there. Further, for DE, it appears that all nondiagonal matrix elements of the Jacobian are zero, as $s_{ij}=\pm 1$ for each link. The eigenvalues are equal just to the diagonal matrix elements as follows:

(3) $$\lambda_{ij}=-2s_{ij}\sum_{k}s_{ik}s_{kj}$$

and their negativity in a balanced state is a consequence of the condition of balance of each particular triad ijk. (We note that two indices in λ are necessary because the Jacobian is the matrix for links, not for nodes.) In Figure 1, a set of trajectories is shown: $s_{ij}\left(t\right)$, for N=9. As we can see, the trajectories tend to values ±1. The stable sets of these values are the balanced states. We note that when time is reversed, the system is driven to states where all eigenvalues are positive, i.e. $s_{ij}s_{jk}s_{ki}=-1$. The number of such states is equal to the number of balanced states. In other words, the dynamics with reversed time and all links with reversed sign cannot be distinguished from the original dynamics.

The challenge is to drive an initial state of the system with randomly selected values of links to the balanced state, which is most close to this initial state. The distance $d_{H}$ between states $s_{i}$ and states $s_{f}$ is measured as follows:

(4) $$d\left(s_{i},s_{f}\right)=\frac{1}{L}\sum_{i,j,i>j}\left|s_{ij}^{i}-s_{ij}^{f}\right|/2$$

As the compared states are ±1, this definition is equivalent to the Hamming distance, i.e., just the number of different matrix elements, normalized by the number *L* of links. Note that to calculate the distance, we take $\varepsilon_{ij}=0$.

To identify the balanced configuration minimally distant from $s_{i}$, we check all $2^{N}$ balanced configurations. This is feasible for *N* not larger than, say, 25. To construct the set, we prepare all possible chains $w_{i}=\pm 1$ of length *N*. For such a chain, the relation between nodes *i* and *j* in a balanced state is just the product of *w*s, i.e., $s_{ij}=w_{i}w_{j}$. Ideally, the balanced state most close to the initial state is just the final state obtained by the best of the algorithms. In this case, the obtained value of the distance $d_{H}$ between the final state and the balanced state most close to the initial state is zero.

## 3. Data

The data were collected in 1974–1998 by the National Opinion Research Center. Here, they are taken from Ref. [26]. They are related to opinions in 44 American states (excluding Hawaii, Idaho, Maine, Nebraska, Nevada and New Mexico). The issues are listed below, together with a specification, in brackets, as to what the high score means:

1. Tolerance (more tolerance);
2. Race (less racist);
3. Abortion (more pro-choice);
4. Religiosity (more religious);
5. Homosexuality (more accepting of homosexuality);
6. Public feminism (more accepting of women’s rights);
7. Environment spending (support higher government spending on the environment);
8. Welfare spending (support higher government spending on welfare);
9. Death penalty (support for death penalty).

Most opinions are coded as real numbers in the scale (0,1), except abortion (0,6) and feminism (0,2). As we are interested in the Pearson correlations, these differences are irrelevant. The data in [26] contain also the positions of the issues in ranks, which are not analyzed here.

The matrix elements r(i,j) of Table 1 are used as the initial values for the time evolution of the relations, given by Equation (1). To this purpose, the diagonal elements r(i,i) are set to zero. We define also the matrix of signs of *r*: a(i,j)=sign(r(i,j)).

## 4. Results

**Figure 2:**
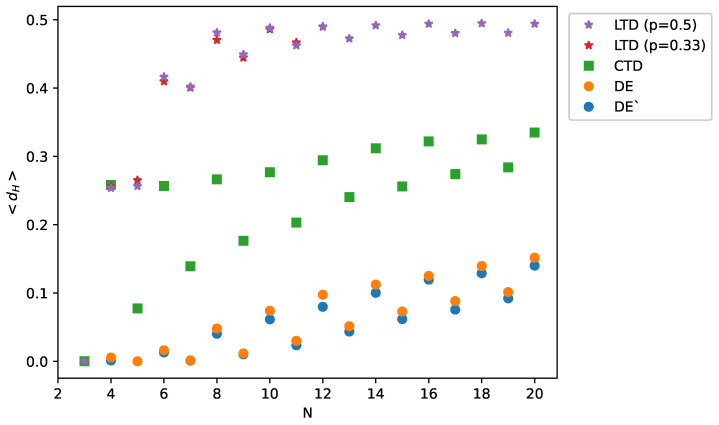
The mean distance $<d_{H}>$ between a final state and a balanced state most close to the initial state, as obtained by particular algorithms, against the number *N* of nodes.

In Figure 2, we show the data on the mean distance $<d_{H}>$ between the final (balanced) state and this balanced state that is most close to the initial state, for all four algorithms: LTD, CTD, DE and DE’. To make these plots, we need to fix three parameters: the probability *p* for LTD, the initial fraction of positive links, and ε for DE. The initial fraction of positive links is set to be 0.5 to ensure maximal diversity of states. When this initial fraction is between 0.4 and 0.6, the results are approximately constant. The values of ε are selected randomly from the uniform distribution between 0.1 and 0.3, separately for each link. Again, in this range of ε, the results do not vary. The probability *p* is set to be 0.3 or 0.5; above the value 0.5, all links are expected to be positive [18]. For all algorithms, the results are averaged over 2000 initial configurations for each value of the number of nodes *N*. The exception is LTD for p=0.33, the case that is particularly time consuming [18], where the statistics is 1000.

The results presented in Figure 2 indicate that the mean distance $<d_{H}>$ between a final state and a balanced state most close to an initial state is shorter when the system is driven by the deterministic algorithms DE and DE’ than in the case of the algorithms LTD and CTD. In other words, DE and DE’ better reproduce the initial state of the system. We calculated also the probability that the final state obtained by each algorithm drives the system exactly to the state most near to the initial state. These results, shown in Figure 2, allow to conclude that the initial state is better reproduced by the deterministic algorithms DE and DE’. The results can be interpreted as a consequence of the randomness of the Monte Carlo algorithms LTD and CTD. On the other hand, the dynamics of DE and DE’ reveal seemingly non-ordered behavior [30,32]—an example is shown in Figure 1—and therefore, the above test is advisable. We also note that in most cases, the differences between the results of DE and DE’ are minimal.

**Figure 3:**
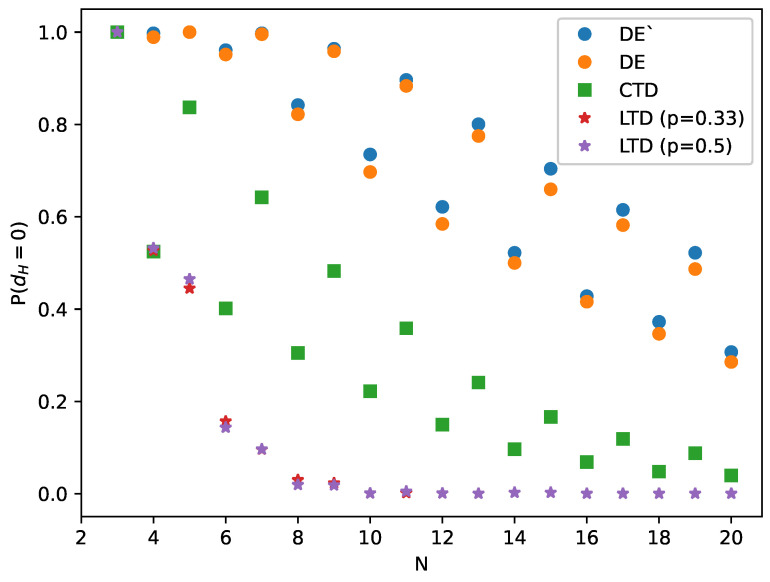
The probability $P(d_{H}=0)$ that the final (balanced) state obtained by particular algorithms is just the same as the balanced state most close to the initial state is dependent on the number of nodes *N*.

If we treat the number *F* of imbalanced triads as a work function, each balanced state is at a local minimum of *F* with the same value F=0. To pass from one minimum to another is equivalent to shifting a node from one group to another, which means that all N−1 links connecting this node to other nodes change their signs. Indeed, we observe that a minimal distance between the nearest-to-initial state and next-to-nearest state (not shown here) is N−1. On the other hand, the effectiveness of DE (and of any other algorithm) decreases with *N*. This is observed in Figure 2 and in particular in Figure 3, where we see that the probability of reaching the distance zero between the final and closest-to-initial state decreases with the number of nodes *N*. This is an indication that the boundaries in the *L*-dimensional space of states between basins of attraction of particular balanced states are more complex for larger *N*.

In Figure 2 and Figure 3, a systematic difference is observed in the performance of all algorithms between even and odd values of *N*; for odd values, the system works better. The origin of this effect is that for even values of *N*, the right side of Equation (1) is sometimes close to zero. This effect slows down the evolution of some links and makes them more susceptible to be shifted to a local minimum of energy *U* that is different from the original one. We note that for LTD, the distance $d_{H}$ reaches 0.5 already for N=8, which means that the correlation between an initial and a final state vanishes.

Now we move to the results of the application of DE and DE’ to the data, described in Section 3. The obtained balanced state most close to the matrix a(i,j) in the sense of the minimal Hamming distance is equivalent to the following partition of the issues: “welfare spending” plus “religiosity” vs. all other issues. When using numbers as in the list of issues given above, the partition is 4,8 vs. 1,2,3,5,6,7,9. On the other hand, the evolution, started from the initial state r(i,j) and driven by both DE and DE’, leads to the balanced state equivalent to the same partition: 4,8 vs. 1,2,3,5,6,7,9. This coincidence indicates that our deterministic algorithms properly identify the split between two groups of statements, collected in [26]. We note that, as the matrix r(i,j) is real and not integer, the difference between even and odd values of *N* is less important.

## 5. Discussion

The advantage of the deterministic algorithms DE and DE’ over the Monte Carlo methods of having a more faithful reproduction of the initial state allows to expect that they could also be successful in a reproduction of a state that is initially balanced and then subject to a noise. This application adds the differential equations DE and DE’ to the list of methods of identification of communities [33], with the specific condition that the number of communities is not larger than two. This condition is a consequence of the duality “friendly–hostile”, imposed by the sociological aspect of the problem. Up to our knowledge, our extension of the method to communities of statements is original.

The partition “welfare spending” plus “religiosity” vs. all other issues, obtained from DE and DE’, is not accidental. It is consistent with the unfulfilled need of safety, which is basic in the hierarchy of needs [34]. It is also supplementary to the comfort hypothesis, which states that “Parishioners whose life situations most deprive them of satisfaction and fulfillment in the secular society turn to the church for comfort and substitute rewards” (Ref. [35]; see also [36] pp. 107–108). The obtained partition indicates that anticipation of reward in heaven does not exclude the expectation of help from Earthly institutions.

The applicability of the deterministic algorithms DE and DE’ to the data reported in [26] indicates that the concept of Heider balance, usually applied to interpersonal relations, can be extended to sets of opinions, gathered in social surveys. We note that the correlations between answers to particular issues are not mutually bound with any logical relations, imposed by the construction of a survey. Therefore, the method of active data handling, described above, can be useful to identify types of collective opinions and beliefs, which are characteristic for a given society [37,38]. 

## Figures and Tables

**Table 1:** The symmetric matrix r(i,j) of Pearson correlations of opinions on nine issues, collected in Ref. [26]. In this setting, the issues are equivalent to nodes (N = 9).

1	0.907	0.686	−0.551	0.828	0.832	0.337	−0.151	0.163
	1	0.565	−0.443	0.828	0.887	0.414	−0.055	0.296
		1	−0.725	0.776	0.600	0.423	−0.219	−0.052
			1	−0.694	−0.571	−0.201	0.277	0.111
				1	0.760	0.354	−0.174	−0.155
					1	0.296	−0.277	0.266
						1	0.080	−0.140
							1	−0.042
								1

## References

[1] Axelrod R. (1997). The Complexity of Cooperation: Agent-Based Models of Competition and Collaboration.

[2] Castellano C., Fortunato S., Loreto V. (2009). Statistical physics of social dynamics. Rev. Mod. Phys..

[3] Nowak A., Vallacher R. (2019). Nonlinear societal change: The perspective of dynamical systems. Br. J. Soc. Psychol..

[4] Pearl J. (2010). The Science and Ethics of Causal Modeling.

[5] Heywood A. (2013). Politics, Palgrave Macmillan.

[6] Converse P.E. (1964). The nature of belief systems in mass publics. Crit. Rev..

[7] Converse P.E., Dalton R.J., Klingemann H.-D. (2007). Perspectives of Mass Belief Systems and Communication, in the Oxford Handbook of Political Behavior.

[8] Bicchieri C. (2006). The Grammar of Society: The Nature and Dynamics of Social Norms.

[9] Heider F. (1946). Attitudes and cognitive organization. J. Psych..

[10] Cartwright D., Harary F. (1956). Structural balance: A generalization of Heider’s theory. Psychol. Rev..

[11] Abelson R.P., Aronson E., McGuire W.J., Newcomb T.M., Rosenberg M.J., Tannenbaum P.H. (1968). Theories of Cognitive Consistency: A Sourcebook.

[12] Bonacich P., Lu P. (2012). Introduction to Mathematical Sociology.

[13] Festinger L. (1957). A Theory of Cognitive Dissonance.

[14] Aronson E., Cope V. (1968). My enemy’s enemy is my friend. J. Personal. Soc. Psychol..

[15] Kulakowski K. (2007). Some recent attempts to simulate the Heider balance problem. Comput. Sci. Eng..

[16] Gawroński P., Kulakowski K. The Heider balance in human networks. Proceedings of the 8th Granada Seminar.

[17] Krawczyk M.J., del Castillo-Mussot M., Hernandez-Ramirez E., Naumis G.G., Kulakowski K. (2015). Heider balance, asymmetric ties, and gender segregation. Phys. A.

[18] Antal T., Krapivsky P.L., Redner S. (2005). Dynamics of social balance on networks. Phys. Rev. E.

[19] Nishi R., Masuda N. (2014). Dynamics of social balance under temporal interaction. EPL.

[20] Harary F. (1961). A structural analysis of the situation in the Middle East in 1956. J. Confl. Resolut..

[21] Moore M. (1979). Structural balance and international relations. Eur. J. Soc. Psychol..

[22] Axelrod R., Bennett D.S. (1993). A landscape theory of aggregation. Br. J. Political Sci..

[23] Lai D. (2001). Alignment, structural balance, and international conflicts in the Middle East, 1948–1978. Confl. Manag. Peace Sci..

[24] de Tocqueville A. (1995). Recollections: The French Revolution of 1848.

[25] Kirkpatrick S., Gelatt C., Vecchi M. (1983). Optimization by simulated annealing. Science.

[26] Brace P., Sims-Butler K., Arceneaux K., Johnson M. (2002). Public opinion in the American states: New perspectives using national survey data. Am. J. Political Sci..

[27] Kulakowski K., Gawroński P., Gronek P. (2005). The Heider balance—A continuous approach. Int. J. Mod. Phys. C.

[28] Antal T., Krapivsky P.L., Redner S. (2006). Social balance on networks: The dynamics of friendship and enmity. Phys. D.

[29] Kulakowski K., Stojkow M., Zuchowska-Skiba D. (2020). Heider balance, stereotyping and size effect. J. Math. Sociol..

[30] Marvel S.A., Kleinberg J., Kleinberg R.D., Strogatz S.H. (2011). Continuous-time model of structural balance. PNAS.

[31] Gawroński P., Kułakowski K., Shi Y., van Albada G.D., Dongarra J., Sloot P.M.A. (2007). A numerical trip to social psychology: Long-living states of cognitive dissonance. Computational Science—ICCS 2007.

[32] Wongkaew S., Caponigro M., Kulakowski K., Borzi A. (2015). On the control of the Heider balance model. Eur. Phys. J. Spec. Top..

[33] Fortunato S., Hric D. (2016). Community detection in networks: A user guide. Phys. Rep..

[34] Maslow A. (1943). A theory of human motivation. Psychol. Rev..

[35] Glock C.Y., Ringer B.B., Babbie E. (1967). To Comfort and to Challenge.

[36] Babbie E. (2007). The Practise of Social Research.

[37] Zaller J.R. (2005). The Nature and Origins of Mass Opinion.

[38] Dornschneider S. (2016). Whether to Kill. The Cognitive Maps of Violent and Nonviolent Individuals.

